# Exploring NS3/4A, NS5A and NS5B proteins to design conserved subunit multi-epitope vaccine against HCV utilizing immunoinformatics approaches

**DOI:** 10.1038/s41598-018-34254-5

**Published:** 2018-10-31

**Authors:** Aqsa Ikram, Tahreem Zaheer, Faryal Mehwish Awan, Ayesha Obaid, Anam Naz, Rumeza Hanif, Rehan Zafar Paracha, Amjad Ali, Abdul Khaliq Naveed, Hussnain Ahmed Janjua

**Affiliations:** 1 Department of Industrial Biotechnology, Atta-ur-Rahman School of Applied Biosciences (ASAB), National University of Sciences and Technology (NUST), Islamabad, Pakistan; 2 Department of Healthcare Biotechnology, Atta-ur-Rahman School of Applied Biosciences (ASAB), National University of Sciences and Technology (NUST), Islamabad, Pakistan; 3 Research Center for Modeling & Simulation (RCMS), National University of Sciences & Technology (NUST), Islamabad, Pakistan; 4 Islamic International Medical College, Riphah International University Rawalpindi, Islamabad, Pakistan

## Abstract

Hepatitis C virus (HCV) vaccines, designed to augment specific T-cell responses, have been designated as an important aspect of effective antiviral treatment. However, despite the current satisfactory progress of these vaccines, extensive past efforts largely remained unsuccessful in mediating clinically relevant anti-HCV activity in humans. In this study, we used a series of immunoinformatics approaches to propose a multiepitope vaccine against HCV by prioritizing 16 conserved epitopes from three viral proteins (i.e., NS34A, NS5A, and NS5B). The prioritised epitopes were tested for their possible antigenic combinations with each other along with linker AAY using structural modelling and epitope–epitope interactions analysis. An adjuvant (β-defensin) at the N-terminal of the construct was added to enhance the immunogenicity of the vaccine construct. Molecular dynamics (MD) simulation revealed the most stable structure of the proposed vaccine. The designed vaccine is potentially antigenic in nature and can form stable and significant interactions with Toll-like receptor 3 and Toll-like receptor 8. The proposed vaccine was also subjected to an *in silico* cloning approach, which confirmed its expression efficiency. These analyses suggest that the proposed vaccine can elicit specific immune responses against HCV; however, experimental validation is required to confirm the safety and immunogenicity profile of the proposed vaccine construct.

## Introduction

Hepatitis C virus (HCV) infected patients are currently estimated to number ~130 million worldwide^1^. Chronic HCV infection leads to 0.88 million deaths annually due to infection-induced liver cirrhosis and hepatocellular carcinoma. Despite decades of research, there is still no effective vaccine available for HCV due to the high genetic heterogenicity of the HCV ribonucleic acid (RNA)^1^. Currently available standard treatments of HCV infection include peginterferon alpha/ribavirin (PegIfn-α-/RBV) and recently introduced direct-acting antiviral (DAA) agents such as sofosbuvir, ombitasvir, paritaprevir ritonavir, and boceprevir^2^. Although the efficacy of DAAs is quite high in comparison with that of PegIfn α/RBV, still, there are limitations with use of the former including high costs, emerging resistant mutants, and the inability to protect patients from relapse^3^. Therefore, the development of an effective and safe vaccine is needed to better control the ongoing worldwide HCV pandemic.

It is believed that 30% of HCV infected patients spontaneously clear HCV infection due to specific and robust host immune responses^4^. This phenomenon occurs in part due to the exposure of neutralizing antibodies and the production of specific T-cell responses (CD8+, CD4+) to HCV proteins. These activated T-cells secrete proinflammatory cytokines (Th1-type) such as interferon-γ (IFN-γ), which is an important antiviral agent against HCV and is related with the decrease in viral load during acute infection^5^. Similarly, the delayed production of these specific antibodies and T-cell responses has been observed in patients with chronic HCV infection^6^. These observations are clearly evidenced in infected humans and chimpanzees that mount an early natural immunity, which ultimately clears the virus. This scenario gives hope for enhancing specific immune signatures and regarding the development of at least a comparatively effective vaccine against HCV^5^.

However, multiple factors such as the high genetic variability of HCV genome and the potential risks of testing killed or live-attenuated vaccine in clinical trials are major hindrances in the development of a successful vaccine against HCV^7^. To overcome such issues, immunoinformatic approaches represent a promising option to identify, design, and propose a conserved yet highly immunogenic multiepitope vaccine against HCV^8^. Immunoinformatics is an interface between experimental immunology and computer science that is used for investigating significant immunological information hidden in the immune system^9^. Previously, immunoinformatic approaches have been successfully employed to develop vaccines that target rapidly mutating infectious diseases^10^. For example, multiepitope vaccines against influenza and human immunodeficiency virus-1 are currently at different stages of clinical trials^11^. In addition, a multiepitope vaccine (EMD640744) designed against advanced solid tumour has also entered phase I clinical trials^12^. In view of these successes, the importance of immunoinformatic approaches in vaccine design is enhanced and become more reliable. Moreover, multiepitope vaccines have significant advantages as compared with conventional vaccines in terms of their safety profile and immunogenic properties, including that they are composed of multiple major histocompatibility complex (MHC) I and II-restricted epitopes recognised by various clones of T-cells^13^. This property enhances their ability to induce strong cellular and humoral immune responses simultaneously. Furthermore, they are composed of some adjuvants that can improve the immunogenicity and immune responses associated with the designed vaccine^12^. Therefore, an increasing amount of research attention has now shifted toward the understanding of an immunoinformatic based multiepitope vaccine design against HCV.

An ideal HCV multiepitope vaccine should include conserved immunogenic epitopes that can elicit effective CD4+, CD8+ T and B-cell responses^14^. Activation of these HCV-specific immune responses is critical for an ideal therapeutic vaccine to induce their recruitment to the liver, where they can deploy their antiviral activity by secreting various cytokines, including more specifically IFN-γ, or by directly killing infected hepatocytes^2^. Thus, safe and HCV-specific immune responses can be induced with improved effectiveness and extent by employing the conserved epitopes together.

Towards achieving this goal, the current study was designed to identify putative T-cell epitopes for multiepitope vaccine design. A comprehensive conservational analysis was carried out among selected viral proteins in HCV major genotypes. In order to design the multiepitope vaccine, T-cell epitopes were selected according to those with the highest probability of being presented by MHC I and MHC II molecules based on affinity prediction score. Afterwards, these selected T-cell epitopes based on their shared sequences with conserved regions, B-cell epitopes, and IFN-γ-binding epitopes were filtered. Different combinations of selected epitopes were anticipated using structural modelling and epitope–epitope interactions analysis to obtain the final effective vaccine construct. In order to enhance the immunogenic response of the vaccine, the amino acid sequence of β-defensin, an important adjuvant, was also added at the N-terminal end of the final vaccine construct. The structure of the proposed vaccine construct was predicted through I-TASSER^15^ and refined through the GalaxyRefine server^16^. The predicted model was further evaluated by PROSA^17^ analysis and Ramachandran plot^18^. Moreover, the predicted model was verified via checking the overall structure errors and molecular dynamics. Molecular docking of the vaccine construct with Toll-like receptor TLR-3 and TLR-8 was performed to inspect various interactions between TLR-3, TLR-8 and the vaccine construct.

## Results

### Conservation profile of hepatitis C virus NS3/4A, NS5A, and NS5B proteins

Conservation analysis is an extensively used method for the prediction of functionally important residues in protein sequences^19^. Using different software packages (section 4, Methods), 23 conserved regions were recognised among the selected (approximately 1,400) protein sequences of HCV NS3/4A segments (Table 1). Analysis showed that the NS5B sequences (1,100) possess 14 conserved regions across all the genotypes. Within high variable sequences of NS5A, only five regions were found to be conserved (Table 1). Using this analysis, a consensus sequence of all major genotypes against each viral protein was also obtained for further B-cell, T-cell and IFN-**γ** epitope prediction.

**Table 1:** Conserved regions in HCV NS3/4A, NS5A and NS5B.

Conserved regions (NS3/4A)	Positions
APITAY	1–6
LLSPRP	126–131
FRAAVC	154–159
LHAPTGSGKSTKVP	202–215
VLVLNPSVAATLGFG	225–239
TYSTYGKFLADGGC	266–279
IICDECH	287–293
LGIGTVLDQAETAG	301–314
VLATATPPGS	319–328
GEIPFYG	345–351
KGGRHLIFCHSKKKCDE	360–376
TDALMTG	411–417
TGDFDSVIDCN	419–429
VDFSLDPTF	436–444
PQDAVSR	452–458
QRRGRTGRG	460–468
TPGLPVCQDHL	519–529
VFTGLT	535–540
LSQTKQ	547–552
AYQATVC	562–568
APPPSWD	572–579
GPTPLLYRLG	594–603
TSTWVL	631–636
**Conserved Regions NS5/A**	**Positions**
GTFPIN	86–91
GSQLPC	185–190
RGSPPS	220–225
ASSSASQLSAPSL	227–239
SSMPPLEGEPGDP	421–433
**Conserved Regions NS5B**	**Positions**
KKVTFDR	50–56
TTIMAKNEVF	136–145
PDLGVRVCEK	163–172
YGFQYSP	191–197
YDTRCFDSTVTE	219–230
CGYRRCRASGV	274–284
LVCGDDLV	314–321
FTEAMTRYSAPPGD	339–352
TSCSSNVSVA	364–373
YYLTRD	382–387
ARAAWET	393–399
PVNSWLGNII	404–413
MTHFFS	426–431
YLFNWAV	524–530

### Prediction of novel T-cell (major histocompatibility complexes I and II), B-cell, and interferon-γ epitopes

Using the consensus sequence of each viral protein, nine-mer T-cell epitopes (MHC-I and II) were predicted. Only T-cell epitopes with high scores and high binding capacities to the maximum number of alleles more specifically, to the alleles involved in HCV clearance or protection including DRBl*ll04, DRB*5701, DRB*5703, DRB1*0701, DQBl*O301, HLA-A*03, DQA*0201, HLA-B*57, HLA-A*68, DRB1*0101, Cw*0102, and HLA-B*27 were screened^20–22^. Further, 20-mer B-cell and 15-mer IFN-**γ** epitopes were predicted against each viral protein. T-cell epitopes overlapping with predicted B-cell and IFN-**γ** epitopes and which were also present in the conserved region were separated, thus designating them as pan-genotypic epitopes. The predicted epitopes were evaluated by BLASTp to avoid epitopes homologous with human proteins. None of the screened epitopes were found to be homologues of human proteins. Antigenicity of the final epitopes was predicted to select only those epitopes that showed high antigenicity. It was observed that most of the selected epitopes exhibited high antigenicity, a finding which confers the importance of these epitopes. A few epitopes with low antigenicity were not considered for further analysis (Table S1). Based on the rigorous selection criteria, the prioritised T-cell epitopes possessed following characteristics: (1) T-cells having a high score and binding capacity to the maximum number of alleles (including alleles causing HCV protection); (2) overlap with B-cell and IFN-**γ** epitopes and presentation in the conserved regions; and (3) high antigenicity and nonhuman homologues. Using these criteria, only 16 epitopes were prioritized for further consideration (Table 2). Among these epitopes, T1 to T8 were present in NS34A, E1 and E2 were present in NS5A, while M1 to M5 existed in the NS5B region (Table 2).

**Table 2:** Selected 16 T-cell (MHCI and II) epitopes among HCV NS3/4A, NS5A and NS5B proteins.

Epitopes (MHCI) NS3/4A	Name	Position	Antigenicity	Epitopes (MHCII) NS3/4A		Position	Antigenicity
GSGKSTKVP	T1	207	0.7	VLNPSVAAT	T4	226	1.2
LNPSVAATL	T2	228	0.9	LNPSVAATL	T5	227	0.9
HSKKKCDEL	T3	368	0.9	LGIGTVLDQ	T6	300	0.6
				IFCHSKKKC	T7	365	1.5
				FCHSKKKCD	T8	366	1.3
**Epitopes (MHCI) NS5A**	**Name**	**Position**	**Antigenicity**	**Epitopes (MHCII) NS5A**		**Position**	**Antigenicity**
				SSMPPLEGE	E1	421	1
				SMPPLEGEP	E2	423	1
**Epitopes (MHCI) NS5B**	**Name**	**Position**	**Antigenicity**	**Epitopes (MHCII) NS5B**		**Position**	**Antigenicity**
TTIMAKNEV	M1	136	0.8	TIMAKNEVF	M4	137	0.6
DLGVRVCEK	M2	164	0.9	LGVRVCEKM	M5	164	0.6
YDTRCFDST	M3	219	1.1	YRRCRASGV	M6	275	1.2

### Design of multiepitope vaccine

**Figure 1:**
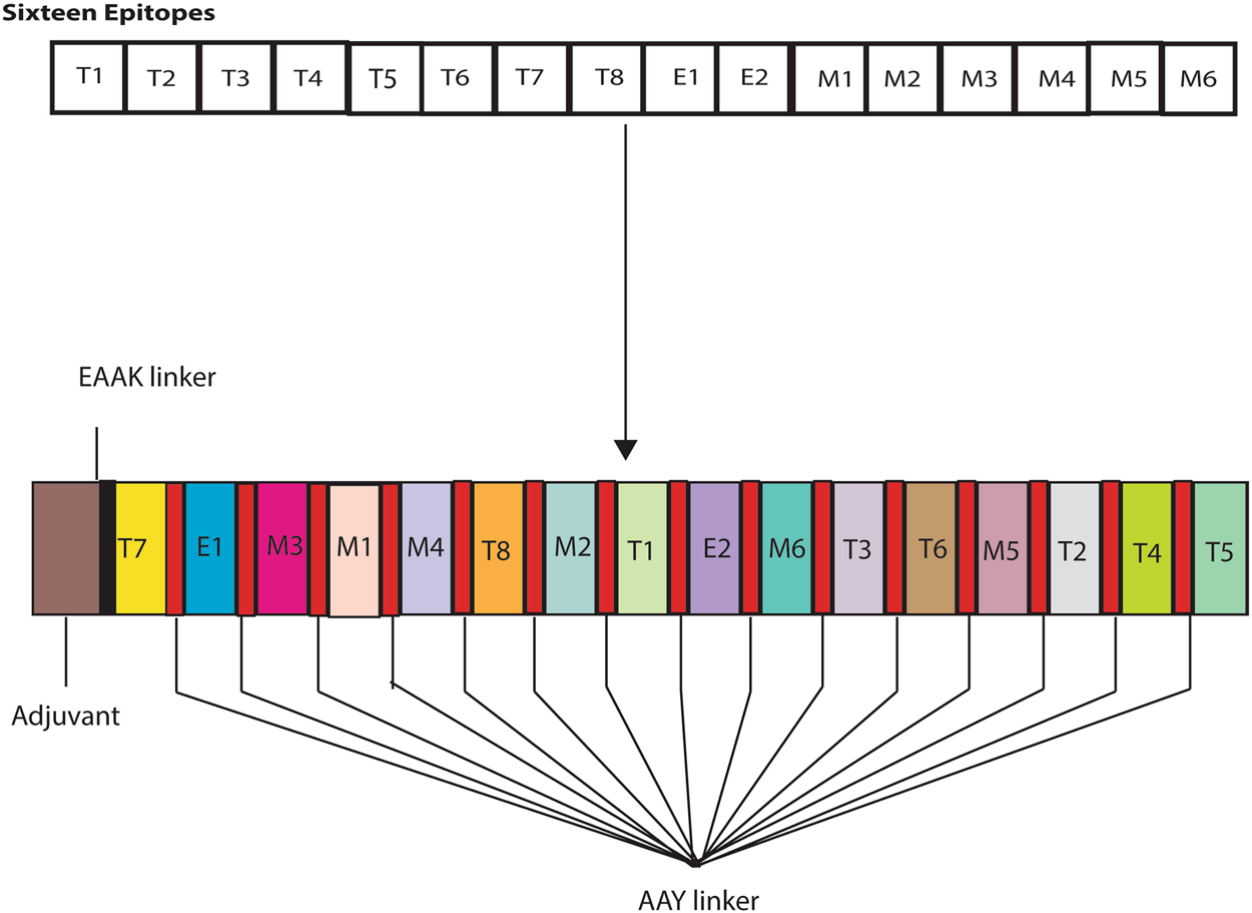
Schematic illustration of vaccine construct consists of epitopes joined together by linkers and an adjuvant. Epitopes combinations were obtained after checking their binding compatibility with each other through schematic HADDOCK refinement score.

The epitopes mentioned in Table 2 were analysed for their binding compatibility with one another. For this, their structures were first predicted with a flexible linker AAY using I-TASSER. Epitopes in combinations of two were then subsequently explored to identify the most promising structure for an active and stable candidate (Fig. 1). The T7-E2 combination was found to be the best option, based on the HADDOCK refinement scores of all of the combinations tested (Supplementary File 1). The T7-E2 combination was explored with the remaining 14 epitopes to secure the three best epitope combinations. In this analysis, the T7-E2-M3 combination was prioritized based on the determined scores (Fig. 1). The final vaccine construct obtained through this extensive combination analysis was T7-E2-M3-M1-M4-T8-M2-T1-E3-M6-T3-T6-M5-T2-T4-T5 (Table 3). Each possible combination prioritized for vaccine construct along with their features is shown in Table 3. Also, adjuvant β-defensin of a 45-amino-acid-length (GIINTLQKYYCRVRGGRCAVLSCLPKEEQIGKCSTRGRKCCRRKK) was added at the N-terminal of the vaccine construct with the help of another linker (EAAAK). With the addition of the adjuvant and linkers, the final length of the vaccine candidate was 239 amino acids.

**Table 3:** Best epitopes combinations based on structural modeling and epitope-epitope interactions analysis.

Best epitopes combinations	HADDOCK refinement score
T7-E1	−81.8 +/− 1.7
T7-E1-M3	−93.1 +/− 5.9
T7-E1-M3-M1	−116.7 +/− 1.8
T7-E1-M3-M1-M4	−107.3 +/− 1.1
T7-E1-M3-M1-M4-T8	−105.1 +/− 2.5
T7-E1-M3-M1-M4-T8-M2	−124.9 +/− 0.9
T7-E1-M3-M1-M4-T8-M2-T1	−91.9 +/− 1.1
T7-E1-M3-M1-M4-T8-M2-T1-E2	−109.8 +/− 2.5
T7-E1-M3-M1-M4-T8-M2-T1-E2-M6	−96.3 +/− 0.9
T7-E1-M3-M1-M4-T8-M2-T1-E2-M6-T3	−93.1 +/− 4.2
T7-E1-M3-M1-M4-T8-M2-T1-E2-M6-T3-T6	−90.0 +/− 2.8
T7-E1-M3-M1-M4-T8-M2-T1-E2-M6-T3-T6-M5	−91.7 +/− 1.3
T7-E1-M3-M1-M4-T8-M2-T1-E2-M6-T3-T6-M5-T2	−90.9 +/− 2.1
T7-E1-M3-M1-M4-T8-M2-T1-E2-M6-T3-T6-M5-T2-T4	−100.8 +/− 2.7
T7-E1-M3-M1-M4-T8-M2-T1-E2-M6-T3-T6-M5-T2-T4-T5	−90.9 +/− 1.3

### Physiochemical properties of the vaccine candidate

Several physicochemical properties of the predicted vaccine candidate were calculated from the ProtParam server^23^. The molecular weight of the vaccine construct was calculated to be 25802.95 g/mol, while the PI was 9.26, identifying it as basic in nature. Furthermore, the instability index (II) was computed to be 39.19, which signifies it is a stable protein complex. The estimated half-life of the vaccine candidate was determined to be 30 hours in mammalian reticulocytes (*in vitro*), while it is predicted to be >20 hours in yeast (*in vivo*) and >10 hours in *Escherichia coli* (*in vivo*). The aliphatic index was 72.09, pointing out the construct as thermostable. The grand average of hydropathicity (GRAVY) score was −0.1108, which shows that, overall, the protein is hydrophilic in nature and can yield better interaction with neighbouring water molecules.

### Modelling of vaccine three-dimensional structure

**Figure 2:**
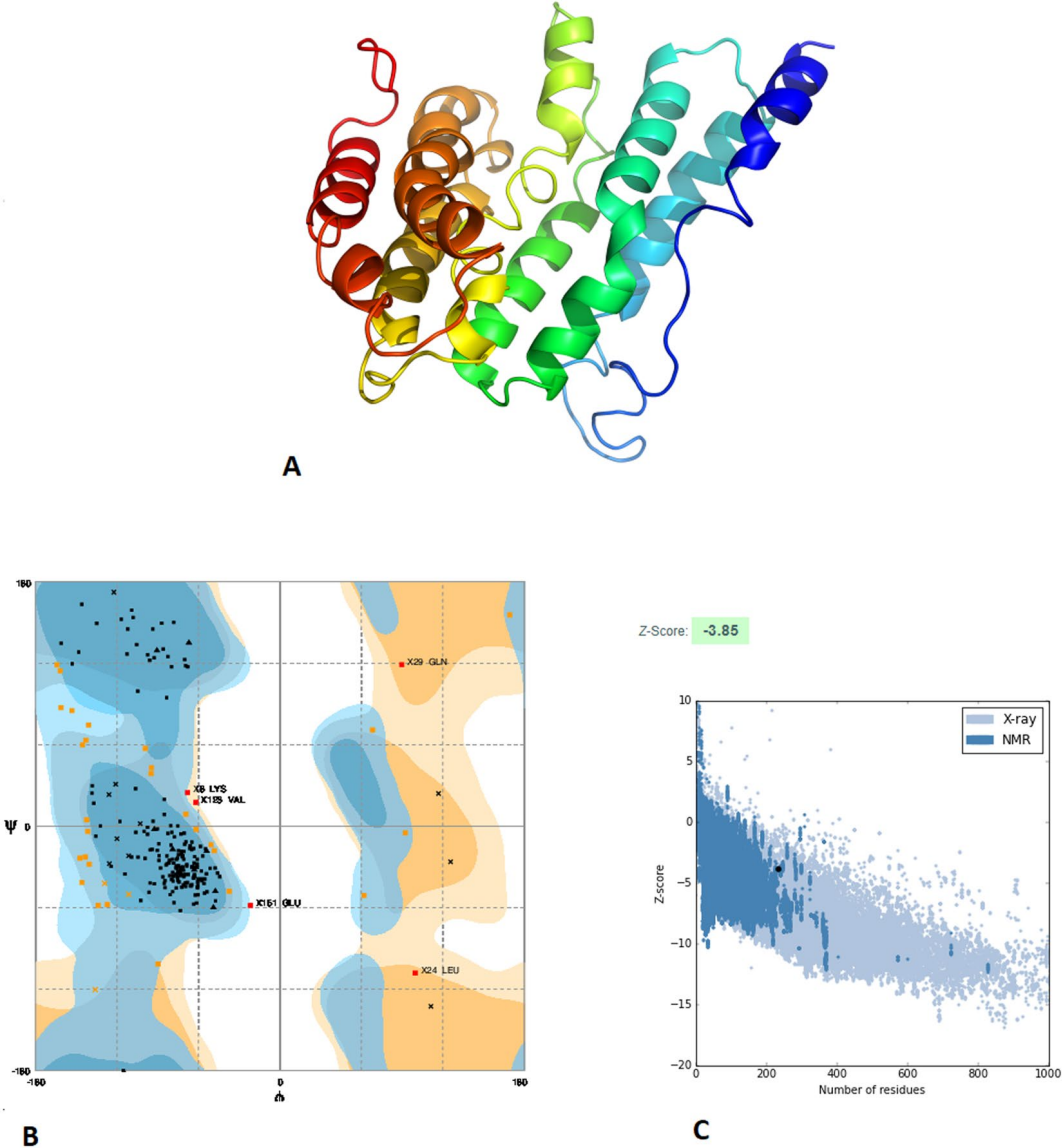
Predicted 3D structure and validation of multiepitope vaccine construct. (**A**) 3D structure of vaccine construct. (**B**) Ramachandran plot showed that 84.6% of the proposed vaccine construct residues were present in favoured regions, 13.3% in allowed regions while only 2.1% in disallowed regions. (**C)** PROSA validation of 3D structure showing Z-score (−3.85). The *z*-score indicates overall model quality.

The three-dimensional (3D) structure of the final vaccine construct was modelled by use of the I-TASSER server^15^. The model was predicted using PDB ID 1kj6 as the best template for modelling, and all 239 amino acids were modelled (Fig. 2). The confidence of the model predicted by iTASSER was quantitatively measured by C-score. It is intended based on the significance of threading template alignments and convergence parameters of the structure assembly simulations. The C-score of the predicted model was higher (−2.20), which represents a model with higher stability and confidence.

### Refinement and validation of the vaccine three-dimensional structure

The predicted 3D structure was then processed to obtain a refined model. From use of the GalaxyRefine server^24^, the best model was chosen for further analysis. The RAMPAGE server^18^ was used for the validation of the refined tertiary structure. This analysis showed that 84.6% of the structure was under favoured region, 13.3% was under the allowed region, and 2.1% was observed under the disallowed region, signalling a high quality of the predicted vaccine structure (Fig. 2). Additionally, ProSA-web showed a Z-score of −3.85, which lies inside the range of acceptable scores.

### Secondary structure prediction

**Figure 3:**
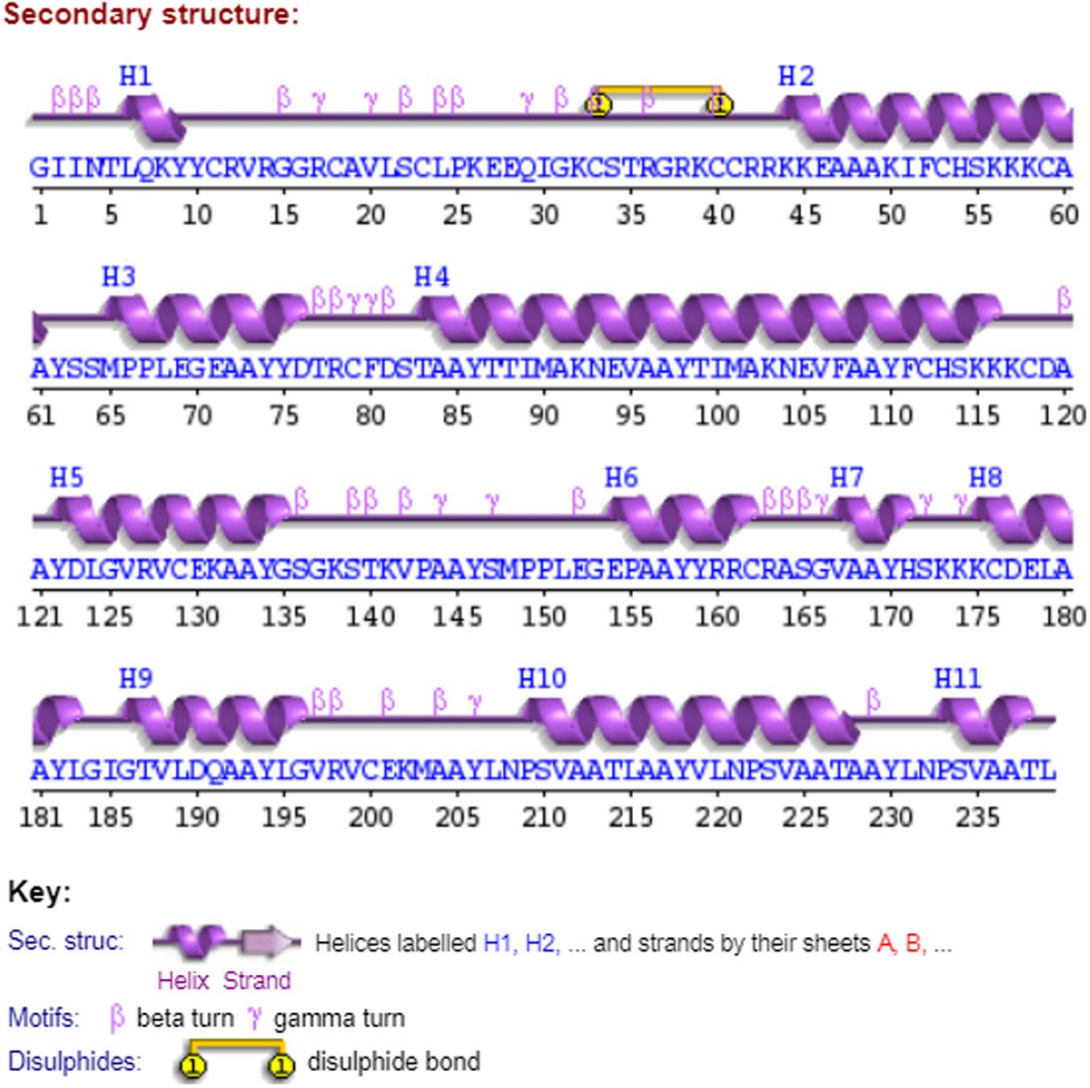
Schematic illustration of secondary structure prediction of vaccine construct.

The secondary structure of the vaccine construct showed that the vaccine construct contained 11 helices, 22 helix–helix interactions, 28 beta turns, 11 gamma turns, and one disulphide bond (Fig. 3, Supplementary File 2). Helix–helix interaction provides information about interacting pairs of helices in the protein. Beta turn is defined for four consecutive residues (denoted by i, i + 1, i + 2, and i + 3) if the distance between the Calpha atom of residue i and the Calpha atom of residue i + 3 is less than 7 Å and if the central two residues are not helical. A gamma turn is defined for three residues i, i + 1, and i + 2 if a hydrogen bond exists between residues i and i + 2 and the phi and psi angles of residue i + 1 fall within 40 degrees of one of the following two classes: (1) classic [phi i + 1(75), psi i + 1(−64)] or (2) inverse [phi i + 1(−79), psi i + 1(−69)]. While disulphide bridges are recognised between two cysteine residues whose sulphur atoms are less than 3 Å apart. The topology of the vaccine construct was generated and analysed, as shown in Fig. 3, Supplementary File 2.

### Molecular dynamics simulation of vaccine construct

**Figure 4:**
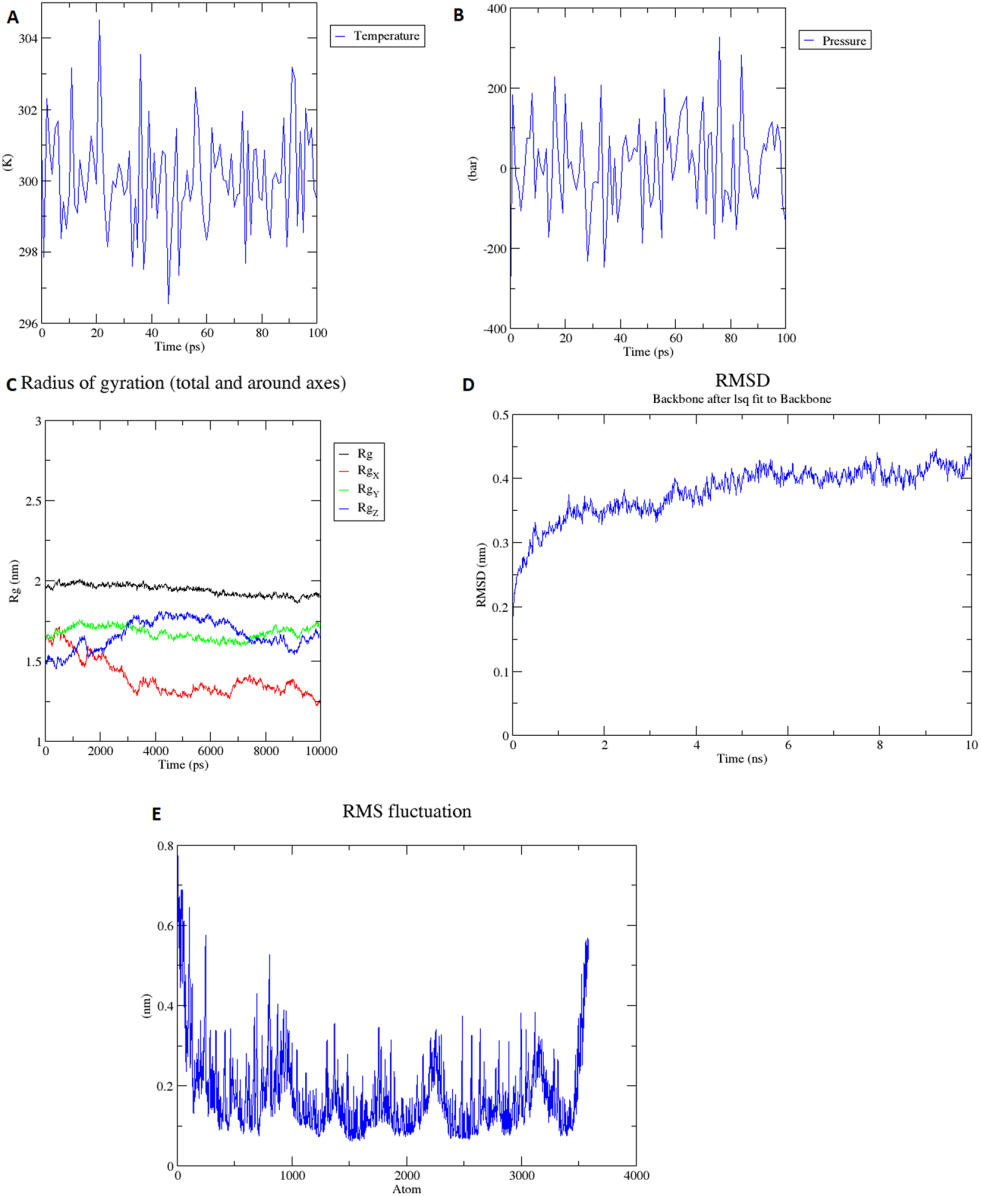
Molecular dynamics simulation of the vaccine candidate (**A**) Temperature; system temperature reached to 300 K and stay constant around 300 K (100 ps) (**B**) Pressure; Ligand pressure plot at equilibration phase of 100 ps. (**C**) Rg plot; vaccine construct is stable in its compact form during the simulation time (**D**) RMSD; RMSD levels off to ~0.45 which represents the stability of vaccine construct (**E**) RMSF; RMSF-Root Mean Square Fluctuation plot, peaks shows the regions with high flexibility.

Energy minimization, pressure, temperature, density, radius of gyration (R_g_), and potential energy calculation were also performed or calculated for the final vaccine construct. The overall structure of the protein remained stable during the MD simulation, which can also be seen in the radius of the gyration graph (Fig. 4). OPLSA force-field was applied and protein was solvated in water using the spectro built-in tool of the Groningen Machine for Chemical Simulations (GROMACS). The charge on the protein was +17, so 17 chlorides ions were added by replacing water molecules at atoms 10091, 21623, 17804, 62648, 63926, 13871, 25058, 6428, 16841, 60002, 14495, 66812, 36452, 39041, 50618, 34997, and 40580. The energy minimization was performed at 50,000 steps, whereas it was performed at 1,612 steps where steepest descents were converged and the force reached <1,000 KJ/mol^−1^. The potential energy was −1.2015219e + 6 and the average potential energy of the system was found to be −1.6503e + 06, with a drift of −124,408. The overall pressure was maintained at an average of 4.58503 with a drift of 8.03 bar, while the temperature was maintained at 299.7 °K (Fig. 4). The overall average density of the system after equilibration was 1,014.58 with drift of 0.98 (kg/m^3^). An analysis of trajectory that was generated after 10 ns simulation generated a radius of gyration for RMSD and RMSF. A plot of RMSD showed that RMSD levels go up to ~0.4 nm and remain between 0.4 nm and 0.45 nm throughout the simulation time, indicating that the structure is very stable (Fig. 4). RMSF also explains that the overall structure of the protein remained stable during the MD simulation (Fig. 4).

### Molecular docking of vaccine construct with Toll-like receptor 3 and Toll-like receptor 8

**Figure 5:**
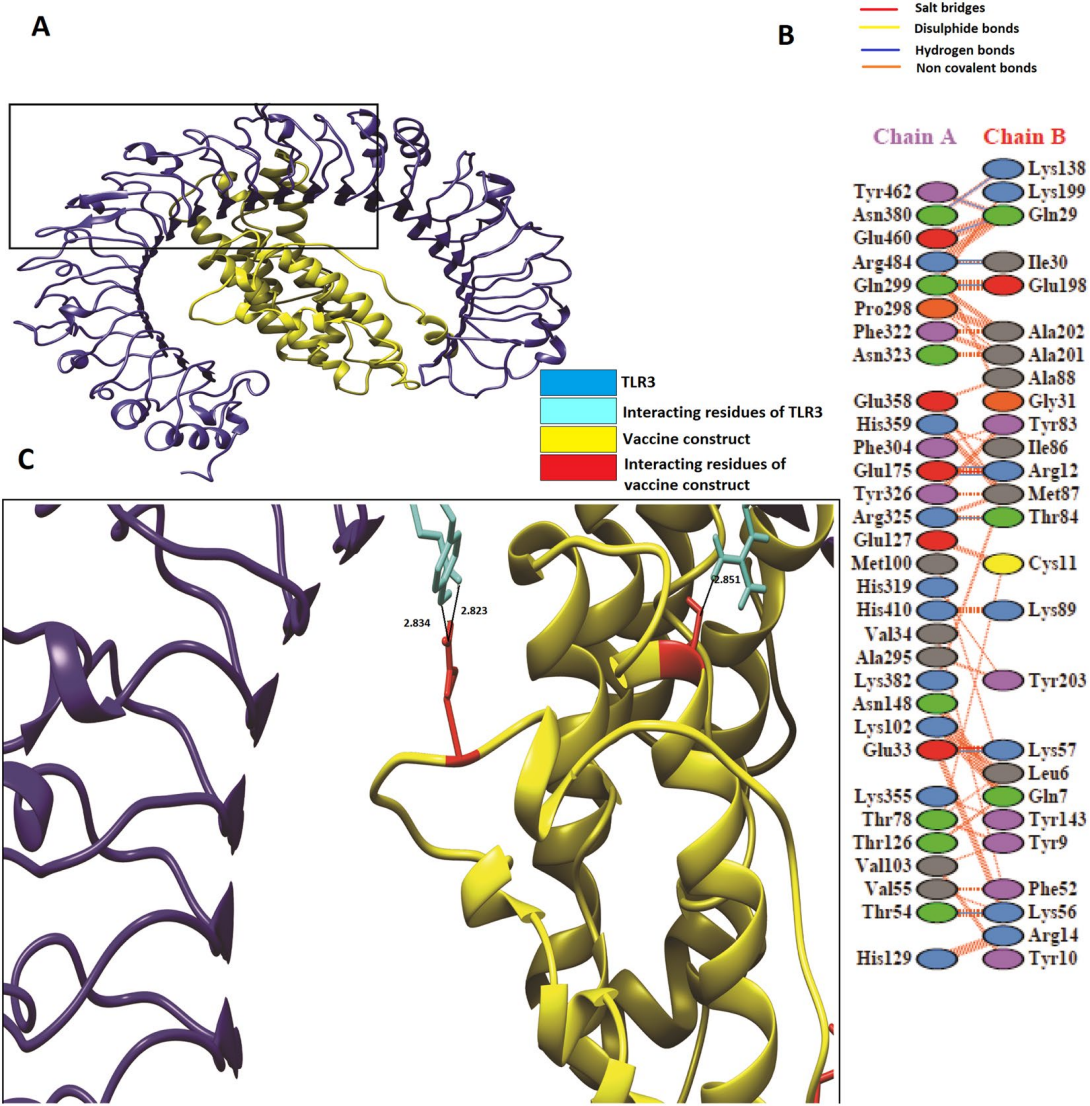
(**A**) Vaccine construct-TLR3 docked complex: Figure obtained after molecular docking between Vaccine and TLR-3, Yellow color showed the vaccine construct while blue color is representing TLR-3. (**B**) Interacting residues illustration between docked vaccine (chain B) and TLR3 (chain A) complex (**C**) Few hydrogen bond interactions between TLR3-vaccine construct are shown in a focused view.

**Figure 6:**
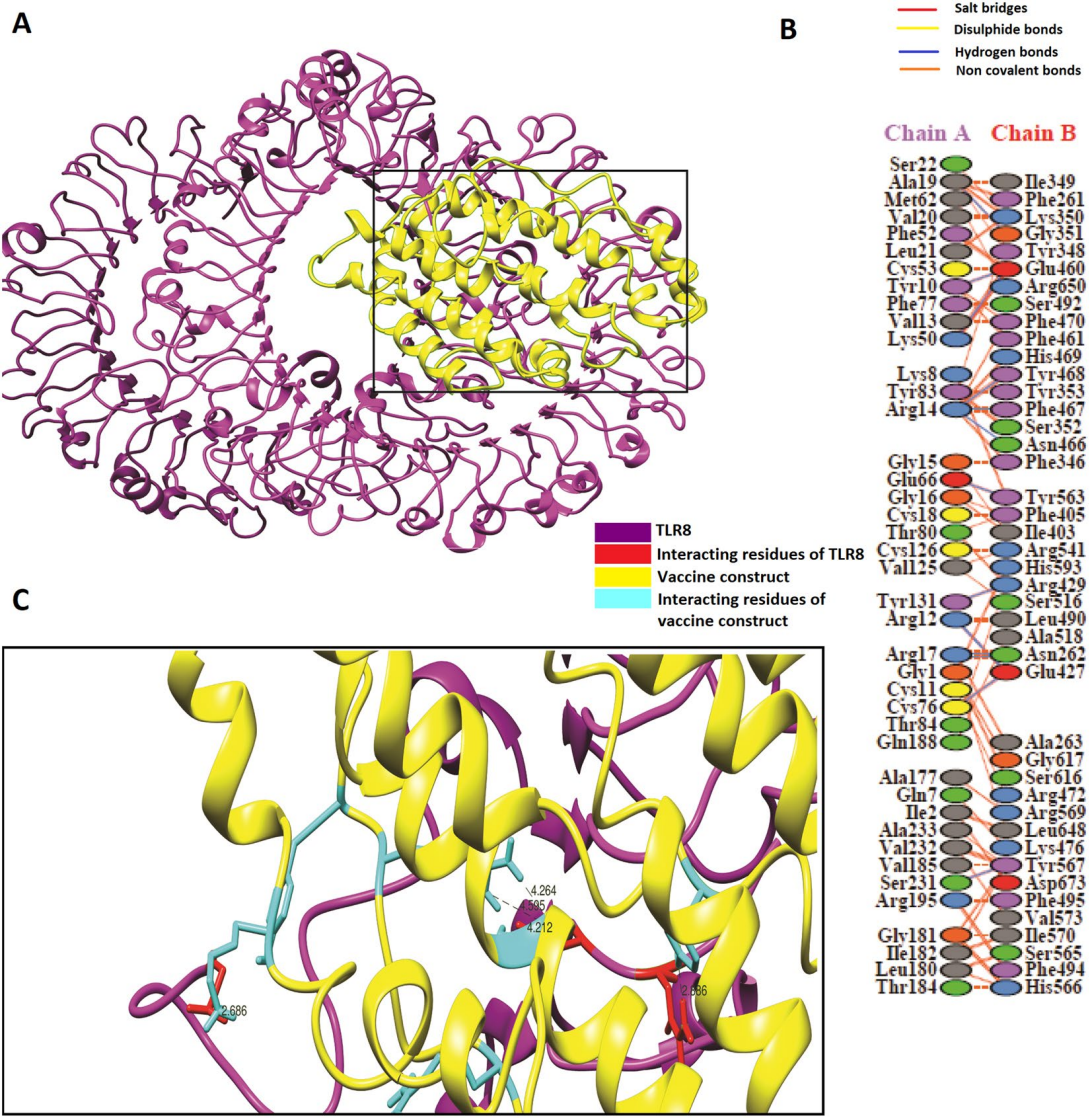
(**A**) Vaccine construct-TLR8 docked complex: Figure obtained after molecular docking between Vaccine and TLR-8, Yellow color showed the vaccine construct while magenta color is representing TLR-8. (**B**) Interacting residues illustration between docked vaccine (chain A) and TLR8 (chain B) complex (**C**) Few hydrogen bond interactions between TLR3-vaccine construct are shown in a focused view.

The HADDOCK server^25^ was used to perform docking analysis of the vaccine construct with TLR3 and TLR8. HADDOCK output was composed of multiple models, out of which the highest 10 clusters were selected. According to HADDOCK, the top cluster is the most reliable, though this ranking is also dependent on its Z-score. A more negative Z-score value represents a better cluster. This analysis also includes fractions of common contacts (FCCs), which are intermolecular contacts and are based on the best HADDOCK model with a cutoff of 5 Å, with interface-RMSD (i-RMSD), which is calculated on the basis of backbone (CA, C, N, O, P) atoms of all amino acid residues, involved in intermolecular contact using a 10 Å cutoff. Finally, ligand-RMSD was calculated on the backbone atoms (CA, C, N, O, P) of all (n > 1) molecules after fitting on the backbone atoms of the first (n = 1) molecule. The docking analysis showed good interaction between the vaccine construct and TLR3/TLR8. TLR3 is shown in the blue colour, while the vaccine construct is shown in the yellow colour, respectively, in Fig. 5. Additionally, TLR8 is shown in the magenta colour and the vaccine construct is shown in the yellow colour in Fig. 6. Furthermore, to obtain the schematic illustration of interaction between the docked complex, the online database PDBsum^26^ was employed. It generated a schematic depiction of nonbonded and hydrogen bond interactions between the docked proteins complex. It was observed that our vaccine construct developed 11 hydrogen bond interactions [Chain A(TLR3)-B(vaccine construct); 33-57, 54-56, 175-12, 175-12, 299-198, 325-84, 380-138, 460-29, 462-29, 484-39, and 484-30] with TLR3. Structural analysis also revealed that Glu33 and Lyc 57 formed a hydrogen bond at a distance of 2.71 Å, while Thr54-Lys56 formed bond at 2.71 Å. Glu175-Arg12 forms two hydrogen bonds at distances of 2.67 Å and 3.32 Å, respectively. Similarly, Gln299-Glu198 develops hydrogen bonds at a distance of 2.89 Å, Arg325-Thr84 at 2.85 Å, Asn138-380Lys at 3.02 Å, Glu460-Gln29 at 2.82 Å, and Tyr29-Gln462 at 2.83 Å, whereas Arg484-Ile39 and Arg484-Ile30 develop hydrogen bonds at distances of 3.08 Å, and 2.99 Å, respectively (Fig. 5, Table S2). In the case of TLR8, it was found that the vaccine construct develops 14 hydrogen bond interactions [Chain A(TLR8)-B(vaccine construct); 460-10, 259-12, 270-14, 293-14, 300-14, 285-17, 292-17, 280-19, 269-50, 261-66, 299-76, 289-131, and 289-231] with immune receptors (Fig. 6, Table S3). It was observed that most of the distances of the hydrogen bonds between the vaccine and immune receptors lie in the range of 2 Å to 3 Å, representing strong interactions^27^. These data also support the evidence that the binding of epitopes to MHC I and MHC II proteins requires multiple hydrogen bond interactions between the epitope and MHC complex^28^ (Figs 5 and 6).

### *In silico* codon optimisation of the vaccine construct

The online server Codon Usage Wrangler was used for reverse translation and to provide a complementary deoxyribonucleic acid (cDNA) sequence. This sequence was further considered for codon optimization analysis, including with respect to codon adaptive index (CAI) and GC content of the cDNA sequence. The GC content of the construct was calculated to be 58.63%, with the ideal range being between 30% and 70%. The CAI value was 1, which signifies high expression of the protein, suggesting the vaccine construct as a reliable protein.

## Discussion

Despite the efficient progress that has been made in the development of antiviral therapies against HCV infection to date, there remains an utmost need to design vaccines in order to halt the inexorable spread of HCV. However, the formation of an HCV vaccine generally represents an unprecedented scientific challenge^2^. Many HCV studies have recognised that a strong T-cell response, categorised by the effective production of cytokines including IFN-γ, is associated with the resolution of acute HCV infection^29^. Reliable induction of robust CD4 and CD8 T-cell responses, similar to what is observed in acute HCV infection, are nowadays under consideration, leading to the recent introduction of studies on advanced vaccine development against HCV^5^. Modern vaccinology based on T-cell epitopes has been successful against some diseases like malaria and cancer and has shown the strongest immunogenicity with regard to eliciting T-cell responses so far^11^. The prevention of HCV chronicity and inhibition of reinfection are the major aims of the present multiepitope T-cell vaccine, which can induce large repertoires of immune specificities; also, notably, it deals with genetic variations effectively, both in pathogens and humans (HLA-specific). Thus, the current research was focused on the designing of a multiple-epitope vaccine that can provide a robust level of protective immunity against HCV. Multiepitope vaccines are known to be more effective than monovalent vacines, as they can induce both cellular and humoral immunities^30^. During acute HCV infection, specific T-cell responses are produced against HCV nonstructural proteins, whereas, during chronic infection, this response drifts toward HCV structural proteins^31^. Therefore, in our study, only nonstructural proteins (i.e., NS3/4A, NS5A, and NS5B) were included in the multiepitope vaccine design to focus on the T-cell reactivity that is related with the acute resolution of HCV. These proteins are also being used in different immunological studies, showing their important status of producing an effective T-cell response, thus making them ideal for multiepitope vaccine design^32^.

Selected T-cell epitopes were found to be conserved among all genotypes and to have high antigenic values, thus defining them as ideal candidates for a pangenotypic vaccine. The conserved and nonhost homologous epitopes have the potential to overcome barriers for epitope-based vaccines and can generate more specific, effective, strong, and long-lasting immune responses while being devoid of all the undesired effects^5^. Previous studies have also confirmed that HCV immune elusion could be circumvented by selecting conserved HCV-specific T-cell epitopes that could target broadly neutralizing antibodies^33^. Predicted T-cell epitopes also overlap B and IFN-γ epitopes and thus can induce both T- and B-cell responses simultaneously. IFN-γ is an important proinflammatory cytokine with known antiviral activity^34^, and vaccine candidates able to activate IFN-γ, inducing T-helper cells, can make them more effective for prompting strong immune responses^35^. Adjuvant was also added in the designed multiepitope vaccines to increase the immunogenicity of the construct and to activate different mediators of innate and adaptive immunity^36^. The physiochemical properties of the designed multiepitope protein predicted it to be stable, hydrophilic, and basic in nature. Various validation studies including Ramachandran plot and ProSA-web indicated that the model is stable in nature.

In order to analyse the immune response of TLR-3 and TLR-8 against our predicted vaccine construct, we performed docking analysis. TLR7 and TLR8 have been found to play critical roles in antiviral immune responses against HCV. Also, recent studies have confirmed that robust TLR3 and TLR8 agonists reduce the level of HCV RNA in HCV-positive patients^37,38^. The predicted RMSD of our refined vaccine construct showed the stable docked complex. In addition, some of the epitopes used in our study for multiepitope vaccine construction were also validated experimentally. In one study, 16 conserved epitopes were reported, of which only 12 epitopes in different combinations reached a population protection coverage level of ≥95%^39^. Among the epitopes predicted by Ibraham *et al*., the epitope HSKKKCDEL in NS3/4A and the epitope TIMAKNEVF in NS5B were similarly predicted in our analysis, conferring the authenticity of the methodology adopted in our study. Furthermore, in another study, CD4+ T-cell epitope LNPSVAATL, predicted as T2 in our study, revealed a high binding affinity for common HLA-DR alleles among patients afflicted with acute HCV infection^40^.

## Conclusion

In summary, we have integrated novel immunoinformatics tools to design a safe and potential immunogenic multiepitope vaccine that could stimulate three types of immune responses—specifically, humoral, innate, and cellular immune responses—and thus may have the ability to control HCV infection. However, this study warrants further experimental validation to prove this work. We expect that our proposed vaccine construct will show promising results against HCV in practice.

## Materials and Methods

**Figure 7:**
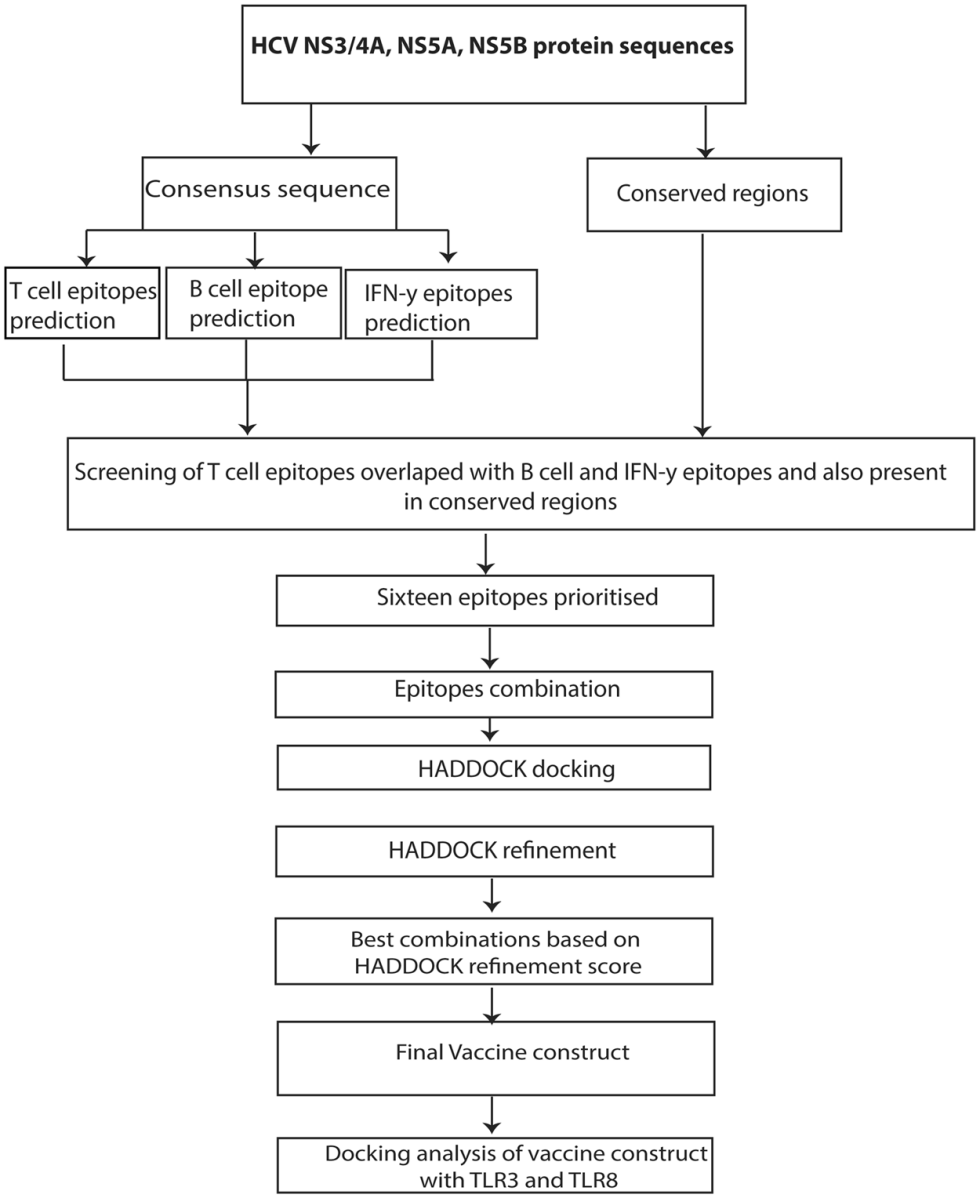
Schematic illustration of methodology adopted in the study.

The present study was categorised into four main parts: (1) the prediction of T-cell epitopes against selected viral proteins (NS3/4A, NS5A, and NS5B); (2) the screening of T-cell epitopes overlapped with predicted B and IFN-γ epitopes and conserved regions; (3) the fusion of selected epitopes by proper linker and adjuvant to propose a multiepitope vaccine construct against HCV by using structural modelling and epitope–epitope interactions based on epitope combinations; and (4) characterising the vaccine construct through molecular dynamics and molecular docking with TLR-3 and TLR-8. An overview of the methods followed in the present study is presented in Figs 1 and 7, and each step is explained below.

### Collection of data

The available protein sequences of NS3/4A (1810), NS5A (1700), NS5B (1350) were retrieved in FASTA format from Genbank^41^. These sequences were selected against all major HCV genotypes (i.e. 1–7). Duplicates were removed by analysing the annotation information of each sequence available from the National Center for Biotechnology Information^42^. Protein sequences having more than two ambiguous amino acids or clones from the same patient were discarded from the dataset.

### Sequence conservation analysis

For sequence conservation analysis, sequences were subjected to multiple sequence alignment (MSA) and position-wise diversity using ClustalW^43^, CLC v3.6 Workbench^43^, BioEdit v. 7.2.3^44^, Datamonkey^45^, and Protein Variability Server (PVS)^46^. The sequences from each genotype were first aligned to obtain the consensus sequence and, subsequently, the achieved consensus sequences were aligned with each other to secure a global consensus sequence and conserved regions among all genotypes (1–7).

### Epitope mapping

By using the consensus sequence of all major genotypes 1 to 7, T-cell (MHC I and II), B-cell, and IFN-γ inducing epitopes were predicted.

#### Major histocompatibility complexes I and II T-cell epitope prediction

Propred I^47^ and Propred^48^ servers were used to predict nine-mer MHC class I and class II epitopes for all of the selected viral proteins. Epitopes that bind to the maximum number of alleles and, in particular, with alleles known to be involved in HCV clearance or protection were selected.

#### Prediction of B-cell epitopes

The prediction of 20-mer linear B-cell epitopes for NS3/4A, NS5A, and NS5B of HCV was achieved using the online immune epitope database and analysis resource available at http://tools.iedb.org/bcell/ ^49^. This computational tool is known for its reliable prediction of linear B-cell epitopes in a given protein sequences.

#### Interferon-γ inducing epitope prediction

IFN-γ is involved in both adaptive and innate immune responses. It also upregulate. opes of HCV, NS3/4A, NS5A, and NS5B against IFN-γ were predicted using the IFNepitope server^50^. This server allows for users to predict and design IFN-γ-inducing peptides having the capacity to induce IFN-γ.

### Comparative analysis with human proteins

Preferably, vaccine candidates should not be human homologues, in order to circumvent autoimmunity. To screen out only nonhuman homologues epitopes, BLASTp^51^ of selected epitopes was performed against human proteome. Epitopes sequences having <30% identity with human proteome were considered to be nonhuman homologues. This analysis was performed to confirm that these epitopes will not activate autoimmunity. Only nonhomologous epitopes were selected for further consideration.

### Screening of antigenic epitopes

T-cell epitopes overlapped with the predicted B-cell and IFN-γ-inducing epitopes and were screened out. Further epitopes present in the conserved regions of all HCV major genotypes were selected. This screening analysis was carried out individually for all selected viral proteins (NS3/4A, NS5A, and NS5B). Antigenicity prediction of selected epitopes was also performed by using the online tool Vaxijen^52^ with the cutoff of a score of ≥0.5. Epitopes having an antigenicity greater than this value were to be considered as antigenic in nature. Antigen prediction by this software is solely based on the physicochemical properties of epitopes using auto cross covariance transformation approach^52^.

### Construction of the multiepitope vaccine

All of the selected epitopes were subsequently analysed for their compatibility to bind effectively with every other epitope in order to determine the order of epitopes in the final vaccine. For this, the following steps were completed:

1. First, a flexible linker AAY^53^ was added in each epitope sequence and their structure was predicted by I-TASSER^15^.
2. Next, each single epitope was analysed for its compatibility with every other epitope using the Guru interface of the HADDOCK server^54^. Clusters with two-epitope combinations having the maximum compatibility of interaction were refined and then evaluated regarding their compatibility with a third epitope. Similarly, clusters with three-epitope combinations showing a maximum compatibility of interaction were then evaluated regarding their compatibility with fourth epitope. The same criteria were carried out in this manner until the final vaccine construct was obtained.

The addition of linkers between two epitopes was completed, as such is helpful in the efficient separation that is required to occur for the effective working of each epitope. Also, the amino acid sequence of β-defensin (which helps in the recruitment of immature dendritic cells and naïve T-cells at the site of infection) was added as an adjuvant. β-defensin was added to the final vaccine construct at its N-terminal with the help of the EAAAK linker.

### Evaluating physicochemical parameters of muti epitope vaccine

To evaluate the physicochemical parameters of multiepitope vaccine construct, including aliphatic index, grand average of hydropathicity, theoretical pI (isoelectrical point), amino acid composition, molecular weight, half-life, instability index, and GRAVY were computed with the ProtParam server^23^ available at http://web.expasy.org/protparam.

### Prediction of secondary structure

For the prediction of the secondary structure and for the purpose of further defining the structural characteristics of the designed vaccine construct, PDBsum^26^ was used. PDBsum is a unique database that shows the molecule(s) that make up the structure of DNA, ligands, proteins, and metal ions as well as the schematic diagrams of their interactions.

### Prediction of tertiary structure of multiepitope vaccine

The tertiary structure of the multiepitope vaccine candidate was predicted from I-TASSER^15^. Such is based on a hierarchical approach to predict protein structure and function. Further refinement of the predicted 3D structure was performed by using the freely available online server GalaxyRefine (http://galaxy.seoklab.org/cgi-bin/submit.cgi?type=REFINE)^16^. This process used both mild and aggressive relaxation methods for refining proteins. It generates many models with more structural deviations from the given structure. For the validation of the vaccine candidate structure, a Ramachandran plot was also created by the use of an online web server called RAMPAGE (http://mordred.bioc.cam.ac.uk/~rapper/rampage.php)^18^. PROSA analysis was performed for further protein structure validation. This resulted in the calculation of an overall quality score for the provided structure. Notably, if this score is outside a specific range for native proteins, then the structure probably contains errors.

### Molecular dynamic simulation of the multiepitope vaccine

To stabilize the structure of the vaccine construct, GROMACS, an MD simulation program, was used. GROMACS is another reliable tool for the study of different biological models within realistic cellular environments^55^. Optimized Potential for Liquid Simulation force-field was selected and the protein was controlled in a rhombic dodecahedron cubic box to accommodate solvent molecules. The protein was placed in the centre of the cube, with the periodic image of the protein being 2 nm apart. The solvent water (spc216.gro) was used to simulate protein, having a force constant (*kpr*) of 1,000 kJ/mol^−1^/nm^−2^. At the place of the solvent molecule, 17 chloride ions were added. Genion (a tool for adding ions within GROMACS) was used to neutralize the overall charges of protein, with a cutoff scheme used (Verlet); also, electrostatic forces were applied. To get the final energy minimized structure, energy minimization was carried out at the 1612 step and the final energy minimised (EM) structure was obtained. The graphs of the stages of energy minimization were analyzsd using Xmgrace^56^. To stabilize the temperature of protein up to a certain value, NVT isothermal-isochoric ensemble equilibration was used at 100 ps. Velocity was generated during NVT equilibration, so that a number of simulations could run at a variety of initial speeds. Using an NPT ensemble consisting of 50,000 steps for the whole process, temperature, pressure, and densities of the stabilized vaccine construct were investigated. MD simulation at 10 ns with 500,000 steps was run for equilibrated construct. The RMSD of backbone of energy minimized were predicted and the results were generated in the form of graphs.

### Docking analysis of the vaccine candidate with immune receptors TLR3 and TLR8

To analyse vaccine molecule interaction with the immune receptors, molecular docking was performed between immune receptors (TLR-3: PDBID 2A0Z and TLR-8: PDBID 4Q0Z) and the multiepitope vaccine candidate. For this high ambiguity-driven, protein–protein docking, the HAADOCK server was used^25^. It is an information-driven flexible docking approach for biomolecular complex modelling. Furthermore, in order to get the schematic illustration of the interactions between docked proteins, the online database PDBsum was utilised^26^. Its analysis includes schematic diagrams of protein–protein interactions.

### *In silico* cloning

The codon usage wrangler server (http://www.mrc-lmb.cam.ac.uk/ms/methods/codon.html) was used for reverse translational analysis. It provides cDNA sequence as an output, which was further analysed for codon optimization, GC content, and CAI by using another online tool called GeneScript^57^. The GC content of a sequence should ideally be present between 30% to 70%, while the ideal CAI score is 1.0, though more than 0.8 can be considered as a good score^57^.

## Electronic supplementary material

Supplementary tables 1,2,3

supplementary file1

supplementary file 2

## Data Availability

All data generated or analysed during this study are included in this article (and its Supplementary Information files).
