# Supplementary material for: Exploring NS3/4A, NS5A and NS5B proteins to design conserved subunit multi-epitope vaccine against HCV utilizing immunoinformatics approaches
Source: Sci Rep. 2018 Oct 31;8:16107. doi: 10.1038/s41598-018-34254-5 (PMC6208421; doi:10.1038/s41598-018-34254-5)
Supplement: Supplementary file 1 — Supplementary tables 1,2,3 [file 41598_2018_34254_MOESM1_ESM.docx]

# **Supplementary Tables**

# **Exploring NS3/4A, NS5A and NS5B proteins to design conserved subunit multi-epitope vaccine against HCV utilizing immuno-informatics approaches**

Aqsa Ikram^1^, Tahreem Zaheer^1^, Faryal Mehwish Awan^1^, Ayesha Obaid^1^, Anam Naz,^1^ Rumeza Hanif^2^, Rehan Zafar Paracha^3^ Amjad Ali^1^, Abdul Khaliq Naveed^4^, Hussnain Ahmed Janjua^1*^

^1^ Department of Industrial Biotechnology**,** Atta-ur-Rahman School of Applied Biosciences (ASAB), National University of Sciences and Technology (NUST),

^2^ Department of Healthcare Biotechnology, Atta-ur-Rahman School of Applied Biosciences (ASAB), National University of Sciences and Technology (NUST)

^3^ Research Center for Modeling & Simulation (RCMS), National University of Sciences & Technology (NUST), Islamabad, Pakistan.

^4^ Islamic International Medical College, Riphah International University Rawalpindi

--------------------------------------------------------------------------------------------------------

Corresponding author: Dr Hussnain Ahmed Janjua*

Department of Industrial Bitechnology, Atta-ur-Rahman School of Applied Biosciences (ASAB), National University of Sciences and Technology (NUST), H-12, Islamabad, 44000 Pakistan

Email: [janjua.hussnain@gmail.com](mailto:janjua.hussnain@gmail.com)

[hussnain.janjua@asab.nust.edu.pk](mailto:hussnain.janjua@asab.nust.edu.pk)

**Table S1:** All predicted T cell epitopes with their predicted antigenicity

| **Epitopes (MHCI) NS3/4A** | **Name** | **Position** | **Antigenicity** |
| --- | --- | --- | --- |
| GSGKSTKVP | T1 | 207 | 0.7 |
| VLVLNPSVA | T2 | 225 | 0.3 |
| LVLNPSVAA | T3 | 226 | 0.4 |
| LNPSVAATL | T4 | 228 | 0.9 |
| TYSTYGKFL | T5 | 266 | 0.1 |
| TVLDQAETA | T6 | 305 | 0.4 |
| HSKKKCDEL | T7 | 368 | 0.9 |
| GPTPLLYRL | T8 | 594 | 0.2 |
| **Epitopes (MHCII) NS3/4A** |  | **Position** | **Antigenicity** |
| LHAPTGSGK | T9 | 202 | 1.0 |
| LVLNPSVAA | T10 | 225 | 0.4 |
| VLNPSVAAT | T11 | 226 | 1.2 |
| LNPSVAATL | T12 | 227 | 0.9 |
| YSTYGKFLA | T13 | 266 | 0.03 |
| YGKFLADGG | T14 | 269 | -0.2 |
| LGIGTVLDQ | T15 | 300 | 0.6 |
| LATATPPGS | T16 | 319 | 0.2 |
| IFCHSKKKC | T17 | 365 | 1.5 |
| FCHSKKKCD | T18 | 366 | 1.3 |
| **Epitopes (MHCI) NS5A** |  | **Position** | **Antigenicity** |
| SSSASQLSA | O1 | 228 | 0.4 |
| ASQLSAPSL | O2 | 231 | 0.4 |
| **Epitopes (MHCI) NS5A** |  | **Position** | **Antigenicity** |
| ASSSASQLS | O3 | 227 | 1.7 |
| SSMPPLEGE | O4 | 421 | 1 |
| SMPPLEGEP | O5 | 423 | 1 |
| PLEGEPGDP | O6 | 425 | 0.9 |
| **Epitopes (MHCI) NS5B** |  | **Position** | **Antigenicity** |
| TTIMAKNEV | M1 | 136 | 0.8 |
| DLGVRVCEK | M2 | 164 | 0.9 |
| YDTRCFDST | M3 | 219 | 1 |
| CGYRRCRAS | M4 | 274 | 0.4 |
| TEAMTRYSA | M5 | 340 | 0.1 |
| TSCSSNVSV | M6 | 364 | -0.1 |
| VNSWLGNII | M7 | 405 | 0.1 |
| **Epitopes (MHCI) NS5B** |  | **Position** | **Antigenicity** |
| TIMAKNEVF | M8 | 137 | 0.6 |
| LGVRVCEKM | M9 | 164 | 0.6 |
| YRRCRASGV | M10 | 275 | 1.2 |
| MTRYSAPPG | M11 | 342 | 1.0 |
| VNSWLGNII | M12 | 404 | -0.3 |

**Table S2:**

**Hydrogen bonds interactions between vaccine construct (chain B) and TLR3 (chain A)**

|  | **Atom no** | **Atom name** | **Residue name** | **Residue no** | **Chain** | **Atom no** | **Atom name** | **Residue name** | **Residue no** | **Chain** | **Distances** |
| --- | --- | --- | --- | --- | --- | --- | --- | --- | --- | --- | --- |
| 1 | 45 | OE2 | GLU | 33 | A | 7123 | NZ | LYS | 57 | B | 2.71 |
| 2 | 233 | OG1 | THR | 54 | A | 7110 | NZ | LYS | 56 | B | 2.71 |
| 3 | 1430 | OE2 | GLU | 175 | A | 6665 | NH1 | ARG | 12 | B | 2.67 |
| 4 | 1430 | OE2 | GLU | 175 | A | 6668 | NH2 | ARG | 12 | B | 3.32 |
| 5 | 2616 | NE2 | GLN | 299 | A | 8440 | GLU | GLU | 198 | B | 2.89 |
| 6 | 2890 | NH1 | ARG | 325 | A | 7374 | THR | THR | 84 | B | 2.85 |
| 7 | 3438 | OD1 | ASN | 380 | A | 7883 | LYS | LYS | 138 | B | 3.02 |
| 8 | 4219 | OE1 | GLU | 460 | A | 6828 | GLN | GLN | 29 | B | 2.82 |
| 9 | 4242 | OH | TYR | 462 | A | 6828 | GLN | GLN | 29 | B | 2.83 |
| 10 | 4470 | NH1 | ARG | 484 | A | 6841 | ILE | ILE | 39 | B | 3.08 |
| 11 | 4473 | NH2 | ARG | 484 | A | 6841 | ILE | ILE | 30 | B | 2.99 |

**Table S3:**

**Hydrogen bonds interactions between vaccine construct (chain B) and TLR8 (chain A)**

|  | **Atom no** | **Atom name** | **Residue name** | **Residue no** | **Chain** | **Atom no** | **Atom name** | **Residue name** | **Residue no** | **Chain** | **Distances** |
| --- | --- | --- | --- | --- | --- | --- | --- | --- | --- | --- | --- |
| 1 | 6029 | O | GLU | 460 | A | 103 | OH | TYR | 10 | B | 2.77 |
| 2 | 5970 | OE1 | GLU | 259 | A | 123 | NH1 | ARG | 12 | B | 4.59 |
| 3 | 5970 | OE1 | GLU | 315 | A | 124 | NH2 | ARG | 12 | B | 4.26 |
| 4 | 6098 | O | PHE | 270 | A | 148 | NH1 | ARG | 14 | B | 3.15 |
| 5 | 6110 | O | TYR | 293 | A | 148 | NH1 | ARG | 14 | B | 2.7 |
| 6 | 6087 | O | ASN | 300 | A | 149 | NH2 | ARG | 14 | B | 2.93 |
| 7 | 4330 | O | ASN | 285 | A | 176 | NH2 | ARG | 17 | B | 3 |
| 8 | 4334 | OD1 | ASN | 292 | A | 176 | NH2 | ARG | 17 | B | 2.85 |
| 9 | 5187 | N | GLY | 280 | A | 194 | OH | ALA | 19 | B | 2.92 |
| 10 | 6033 | OE1 | GLU | 269 | A | 507 | NZ | LYS | 50 | B | 2.69 |
| 11 | 7006 | OH | TYR | 261 | A | 663 | OE1 | GLU | 66 | B | 2.61 |
| 12 | 5970 | OE1 | GLU | 299 | A | 761 | SG | CYS | 76 | B | 2.99 |
| 13 | 5993 | NH1 | ARG | 289 | A | 1283 | OH | TYR | 131 | B | 2.89 |
| 14 | 7051 | TYR | TYR | 289 | A | 2174 | O | SER | 231 | B | 2.89 |

**Supplementary file 1:**

All epitopes combinations with their HADDOCK refinement score (Excel sheet)

**Supplementary file 2:**

Secondary structure prediction details of vaccine construct
